# Health Tract No. 204

**Published:** 1864-05

**Authors:** 


HEALTH TRACT No. 204.

### WHO ARE HAPPIEST?

"MECHANICS' families who are a little forehanded." Such was the answer of a monthly nurse of intelligence and observation, who had in the prosecution of her calling been thrown among families of all classes, from the very rich to the very poor; from the most famed to the most obscure.

Lord Byron seems from his standpoint to have arrived at very nearly the same conclusion. He wrote: "Mechanics and working-men who can maintain their families, are in my opinion the happiest body of men. Poverty is to be preferred to the heartless, unmeaning dissipation of the higher orders."

Another author thought that the most to be envied was "a healthy young man, in full possession of his strength and faculties, going forth in the morning to work for his wife and children, and bringing them home his wages at night."

Aside from the question of religion there are three indispensable requisites to a pleasurable, satisfactory state of the mind; if either be absent, there can not be any continuous mental, heart, enjoyment. In no case can a day ever pass without some interruption to quiet pleasures, even to those who are most favorably situated, because no man or woman ever waked up in the morning who did not experience before retiring at night some disappointment, some unexpected occurrence of an unpleasurable character to cloud the sunshine of the happiest day. Who can recollect a single day in any score or two, or three, in which some unanticipated, disagreeable thing did not occur? Echo answers: "Never one!"

He who would be uniformly happy; who would pass the greater part of his time in a state of mental pleasurableness,

Must be healthy.

Must be well-to-do.

Must be moderately busy.

However healthy a man may be, anxiety for to-morrow's bread will soon undermine the strongest constitution; hence the French returns officially announce that the well-to-do average eleven years longer life than those who live by their daily labor. If a man is healthy and well-to-do, and is not busy in his calling, he will seldom fail to become dyspeptic, intemperate, or restless, and die prematurely. Hence, to have a life of sunshine, a man must live healthfully; must have a reasonably profitable calling, and must be busy and buoyant in the prosecution of it.

